# Ketogenic diet in treating NADH accumulation caused by complex I dysfunction: the metabolic wisdom of “dredging, not repairing”

**DOI:** 10.3389/fped.2026.1939961

**Published:** 2026-10-02

**Authors:** Xiaoying Qiao, Jianxiang Liao

**Affiliations:** Department of Neurology, Shenzhen Children’s Hospital, Shenzhen, China

**Keywords:** complex I deficiency, ketogenic diet, metabolic reprogramming, mitochondrial disease, NADH

## Abstract

Genetic mutations causing Complex I (CI) dysfunction lead to the accumulation of NADH in the mitochondrial matrix due to impaired oxidation. An elevated NADH/NAD⁺ ratio feedback-inhibits key enzymes of the tricarboxylic acid (TCA) cycle, reducing metabolic flux, while concurrent electron transport chain (ETC) blockade exacerbates reactive oxygen species (ROS) generation and oxidative stress. Conventional views propose that the ketogenic diet (KD) bypasses the defective CI to restore downstream ETC electron transfer. However, this perspective harbors an inherent contradiction: CI is the primary entry point for NADH oxidation; bypassing it may sustain some electron flow but fails to consume the accumulated NADH in the matrix, leaving the fate of this critical reducing equivalent inadequately explained. This article proposes the metabolic wisdom of “dredging, not repairing”, positing that KD, through metabolic reprogramming, diverts the accumulated NADH into multiple utilizable pathways. These include intra-matrix conversion and consumption (generating NADPH and ketone bodies), pumping malate from the matrix into the cytosol via the reverse malate-aspartate shuttle (rMAS), and further cytosolic malate utilization (gluconeogenesis, NADPH generation, glycerol-3-phosphate shuttle, etc.). This multi-pathway synergy achieves “dredging”rather than “repair”. This paper aims to elucidate how KD, via metabolic reprogramming, alleviates the inhibition of the TCA cycle caused by NADH accumulation and restores its flux, thereby offering new insights into metabolic therapeutic strategies for CI-deficient mitochondrial diseases.

## 1 Introduction

The ketogenic diet (KD), a metabolic intervention comprising high fat, low carbohydrate, and adequate protein, has demonstrated significant therapeutic potential in treating refractory epilepsy and various mitochondrial diseases (1–3).

Complex I (NADH-ubiquinone oxidoreductase, CI) dysfunction, arising from genetic mutations, represents a common class of mitochondrial diseases (4, 5). Traditional views suggest that KD primarily shifts the electron entry path from CI to Complex II (succinate-ubiquinone reductase, CII) and other routes, bypassing the dysfunctional CI to maintain ubiquinone redox status and downstream ETC electron flux (6, 7). However, analysis of NADH generation pathways (“inflow”) across the three major metabolic routes reveals that matrix NADH accumulates due to impaired CI oxidation. This accumulation further inhibits TCA cycle enzyme activities, exacerbating the “congestion” (8, 9). Conventional explanations lack a clear description of how KD facilitates the re-utilization of accumulated matrix NADH (“outflow”) under energy crisis conditions, which is crucial for restoring TCA cycle flux. Therefore, a systematic exploration of NADH metabolic fates is theoretically and clinically vital for elucidating the mechanisms by which KD ameliorates CI dysfunction from metabolic and energetic perspectives.

## 2 Generation of NADH

Nicotinamide adenine dinucleotide (NAD⁺), composed of nicotinamide riboside, adenine ribonucleotide, and pyrophosphate bridges, is reduced to NADH, a primary electron donor for the mitochondrial ETC (10). NADH generation is intimately linked to the metabolism of three major nutrients (glucose, lipids, proteins) and the TCA cycle (“inflow”). In this paper, based on localization, we distinguish between cytosolic NADH (cNADH), cytosolic NAD⁺ (cNAD⁺), mitochondrial matrix NADH (mNADH), and mitochondrial matrix NAD⁺ (mNAD⁺).

### 2.1 NADH and glucose metabolism

In glucose metabolism, NADH is generated mainly via three processes: glycolysis, pyruvate oxidative decarboxylation, and the TCA cycle.

Glycolysis: Each glucose molecule (Glc) in the cytosol undergoes phosphorylation steps (reactions 1 and 3, Figure 1), consuming 2 ATP. The sixth step, catalyzed by glyceraldehyde-3-phosphate dehydrogenase (GAPDH), oxidizes glyceraldehyde-3-phosphate (GAP) to produce 2 molecules of cNADH (11). Substrate-level phosphorylation in steps 7 and 10 generates 4 ATP. Net yield per glucose: 2 cNADH and 2 ATP.

**Figure 1:**
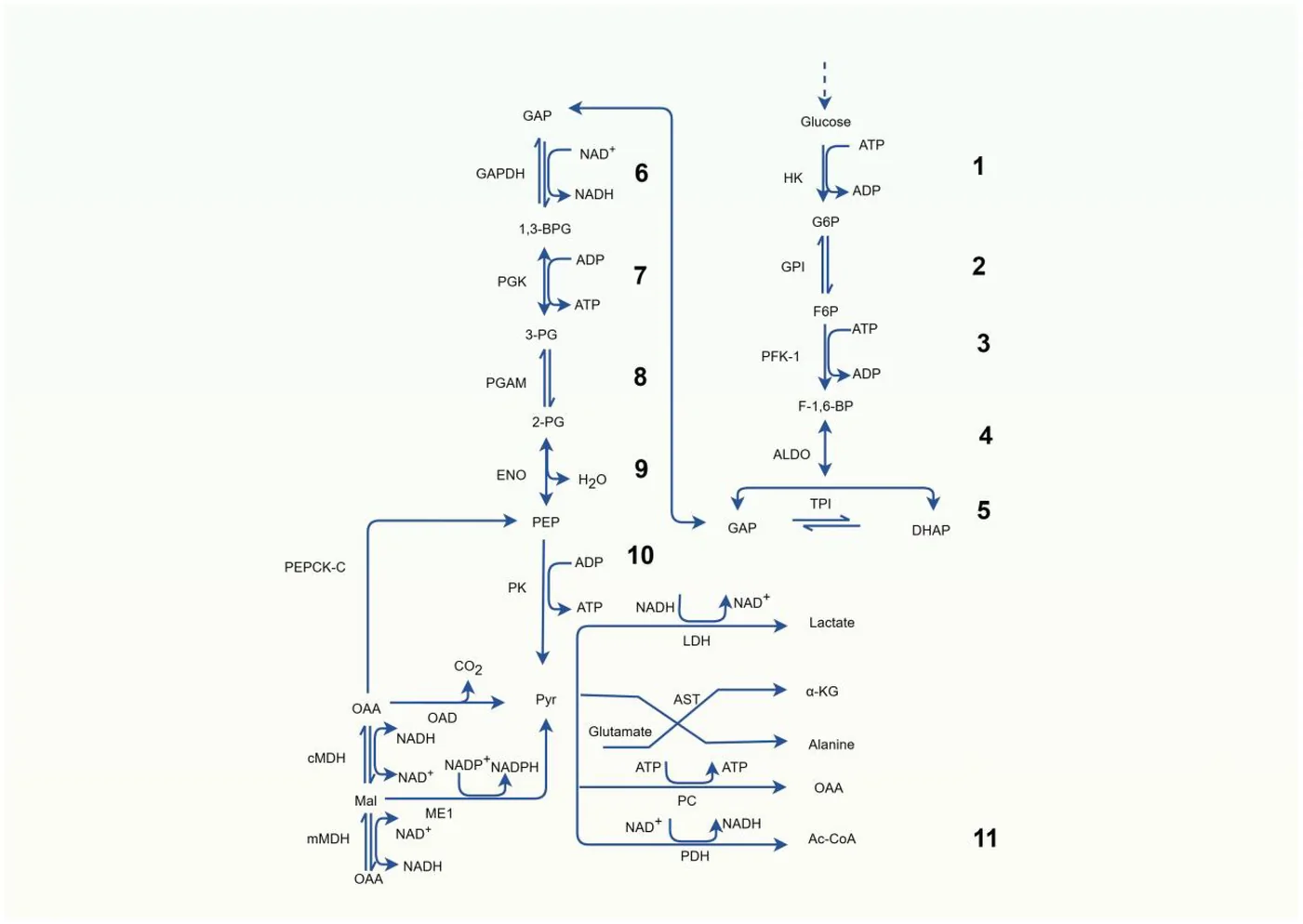
Major NADH-generating processes in carbohydrate metabolism. HK, hexokinase; G6P, glucose-6-phosphate; GPI, glucose-6-phosphate isomerase; F6P, fructose-6-phosphate; PFK-1, phosphofructokinase-1; F-1,6-BP, fructose-1,6-bisphosphate; ALDO, aldolase; DHAP, dihydroxyacetone phosphate; GAP, glyceraldehyde-3-phosphate; TPI, triose phosphate isomerase; GAPDH, glyceraldehyde-3-phosphate dehydrogenase; 1,3-BPG, 1,3-bisphosphoglycerate; PGK, phosphoglycerate kinase; 3-PG, 3-phosphoglycerate; PGAM, phosphoglycerate mutase; 2-PG, 2-phosphoglycerate; ENO, enolase; PEP, phosphoenolpyruvate; PEPCK-C, phosphoenolpyruvate carboxykinase; PK, pyruvate kinase; OAA, oxaloacetic acid; cMDH, malate dehydrogenase; Mal, malic acid; Pyr, pyruvate; ME1, malic enzyme 1; OAD, oxaloacetate decarboxylase; LDH, lactate dehydrogenase; ALT, alanine aminotransferase; *α*-KG, *α*-ketoglutarate; PC, pyruvate carboxylase; PDH, pyruvate dehydrogenase; Ac-CoA, acetyl coenzyme A.

Pyruvate Oxidative Decarboxylation (12): Each pyruvate entering the mitochondrial matrix via the pyruvate transporter (MPC) is converted by the pyruvate dehydrogenase complex (PDH) into one acetyl-CoA and one mNADH (reaction 11, Figure 1). Per glucose, this yields 2 mNADH.

TCA Cycle (13, 14): Each acetyl-CoA in the matrix undergoing one TCA cycle generates 3 mNADH, 1 FADH₂, and 1 GTP (Figure 2). mNADH is produced by isocitrate dehydrogenase (IDH3), *α*-ketoglutarate dehydrogenase (*α*-KGDH), and mitochondrial malate dehydrogenase (mMDH); FADH₂ is produced via complex II (CII). Each TCA turn yields approximately 10 ATP (1 NADH = 2.5 ATP, 1 FADH₂ = 1.5 ATP, 1 GTP = 1 ATP) (15). Per glucose, two TCA cycles produce 6 mNADH, 2 FADH₂, and 2 GTP, totaling 20 ATP.

**Figure 2:**
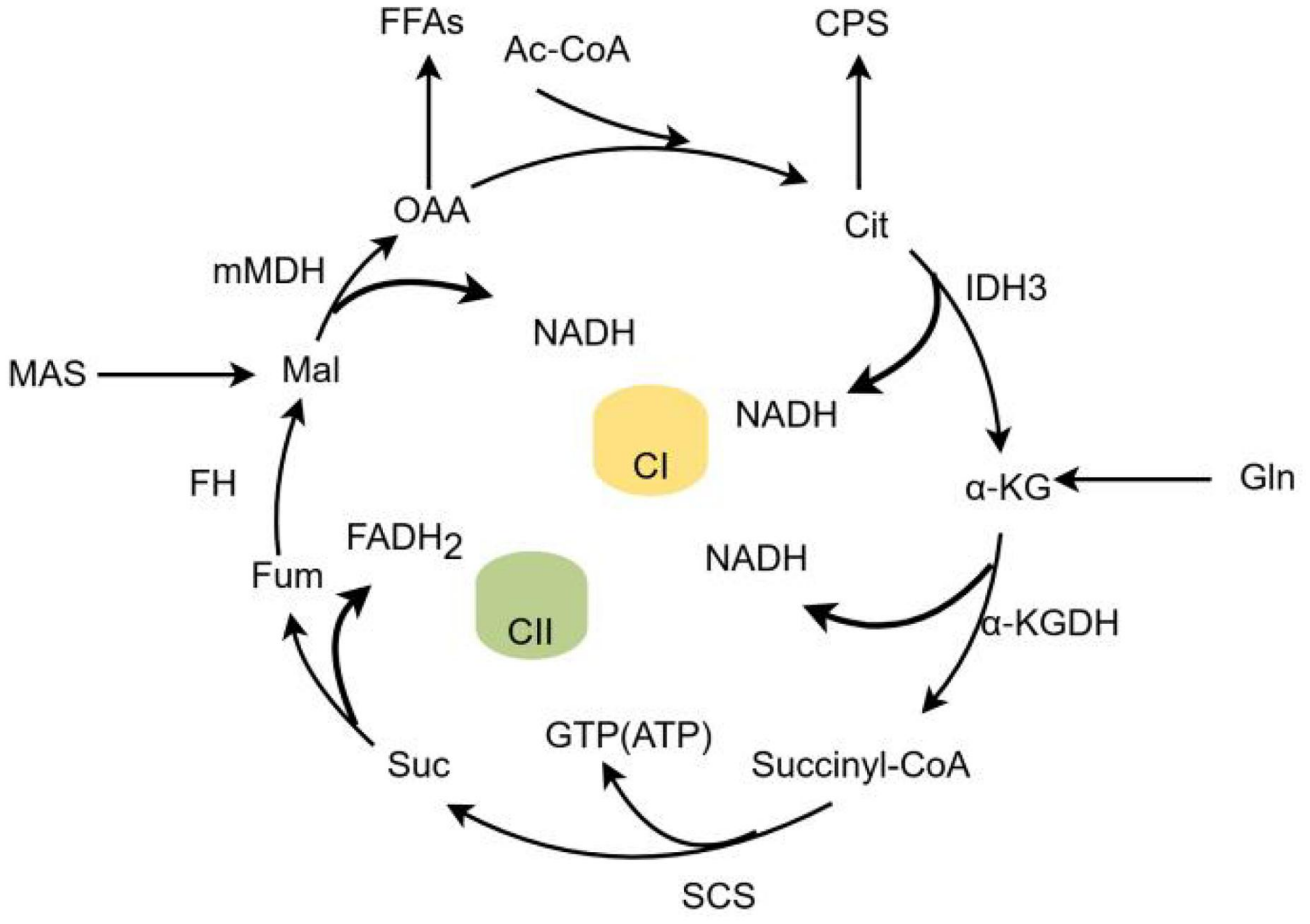
Major NADH-generating steps in the TCA cycle. CPS, citrate-pyruvate shuttle; Cit, citric acid; IDH3, isocitrate dehydrogenase 3; *α*-KG, *α*-ketoglutarate; Glu, glutamate; *α*-KGDH, *α*-ketoglutarate dehydrogenase; Succinyl-CoA, succinyl-coenzyme A; CI, complex I/NADH:ubiquinone oxidoreductase; CII, complex II/succinate dehydrogenase; FH, fumarate hydratase; Mal, malic acid; MAS, malate-aspartate shuttle; OAA, oxaloacetic acid; mMDH, mitochondrial malate dehydrogenase; FFAs, free fatty acids; Ac-CoA, acetyl-CoA; CoQ.

Thus, complete oxidation of one glucose yields 10 NADH (2 cNADH + 8 mNADH), 2 FADH₂, 2 ATP, and 2 GTP, totaling 32 ATP [if 2 cNADH are oxidized via the malate-aspartate shuttle (MAS)] or 30 ATP [if via the glycerol-3-phosphate shuttle (GPS)].

### 2.2 NADH and lipid metabolism

During fasting or KD, an elevated glucagon/insulin ratio activates hormone-sensitive lipase (HSL) via the cAMP-PKA pathway, promoting lipolysis to release free fatty acids (FFAs) and glycerol (16, 17). In lipid metabolism, NADH is generated via mitochondrial fatty acid *β*-oxidation (FAO) and the TCA cycle (13, 18). Ketogenesis in the liver and ketone oxidation in extrahepatic tissues (e.g., brain, heart, kidney) facilitate inter-organ metabolic communication by using ketone bodies as energy substrates (19).

#### 2.2.1 Fatty acid *β*-oxidation

Each round of FAO (Figure 3, reactions 1, 3) yields one FADH₂ (step 1, acyl-CoA dehydrogenase) and one mNADH (step 3, 3-hydroxyacyl-CoA dehydrogenase) (20, 21). FADH₂ transfers electrons to ubiquinone (Q) via the electron-transferring flavoprotein (ETF) and ETF:ubiquinone oxidoreductase (ETF:QO). For an even-chain saturated fatty acid with n carbons, complete oxidation requires (n/2–1) FAO cycles, generating (n/2–1) mNADH and (n/2–1) FADH₂ (21).

**Figure 3:**
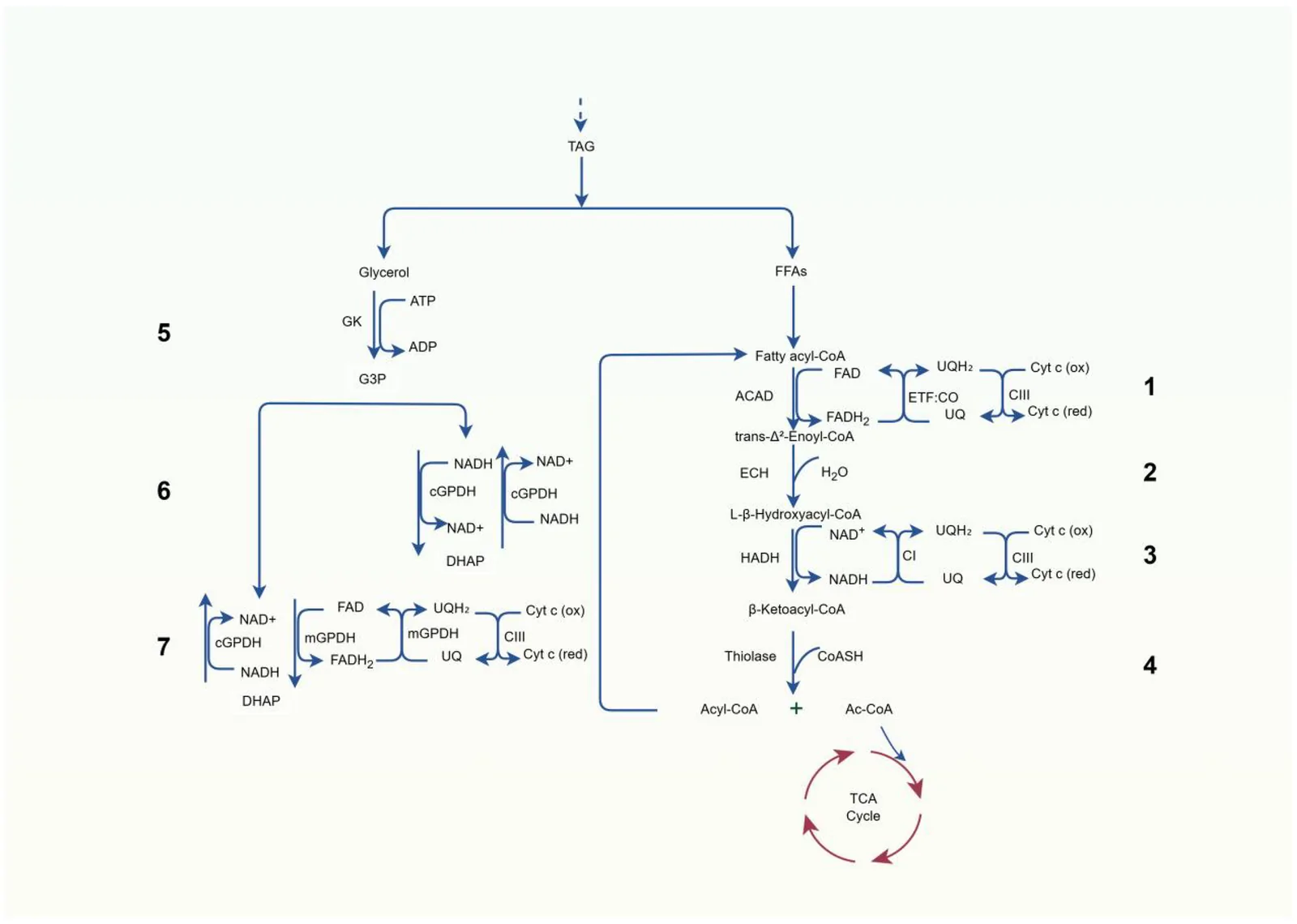
Major NADH-generating processes in triglyceride (TAG) metabolism. TAG, triacylglycerol; FFAs, free fatty acids; ACAD, acyl-CoA dehydrogenase; ETF:QO, electron-transferring flavoprotein-ubiquinone oxidoreductase; UQH₂, reduced ubiquinone; UQ, oxidized ubiquinone; CIII, complex III/ubiquinol-cytochrome c oxidoreductase; Cyt c (ox), oxidized cytochrome c; Cyt c (red), reduced cytochrome c; ECH, enoyl-CoA hydratase; HADH, *β*-hydroxyacyl-CoA dehydrogenase; CI, complex I/NADH:ubiquinone oxidoreductase; Ac-CoA, acetyl coenzyme A; GK, glycerol kinase; G3P, glycerol 3-phosphate; cGPDH, cytoplasmic glycerol-3-phosphate dehydrogenase; DHAP, dihydroxyacetone phosphate; mGPDH, mitochondrial glycerol-3-phosphate dehydrogenase.

Each acetyl-CoA from FAO enters one TCA cycle, generating 3 mNADH, 1 FADH₂, and 1 GTP. For an n-carbon saturated fatty acid (n even), yielding n/2 acetyl-CoA, complete oxidation via FAO and TCA produces (2n–1) mNADH, (n–1) FADH₂, and n/2 GTP. Accounting for the 2 ATP consumed during activation, the total ATP yield is (7n–6). On an equal carbon basis, fatty acids produce far more NADH and FADH₂ than glucose. For 18 carbons: 3 glucose molecules yield 30 NADH (6 cNADH + 24 mNADH) and 6 FADH₂, whereas 1 stearic acid (C18:0) yields 35 mNADH and 17 FADH₂. This higher yield of reducing equivalents stems from direct NADH/FADH₂ generation via FAO and increased acetyl-CoA entry into the TCA cycle.

#### 2.2.2 Glycerol metabolism

Glycerol, the backbone of TAG, is released during lipolysis under energy deficit conditions (fasting or KD) (22–24).

Fate 1: Gluconeogenesis. Primarily in liver and kidney cytosol (25), two glycerol molecules yield one glucose molecule. Glycerol kinase (GK) catalyzes the conversion of glycerol to glycerol-3-phosphate (G3P), consuming ATP; this is the rate-limiting step (26, 27). Cytosolic G3P dehydrogenase (cGPDH) oxidizes G3P to dihydroxyacetone phosphate (DHAP), generating cNADH (reaction 6, Figure 3) (28). DHAP enters gluconeogenesis. Under KD, glycerol is a significant gluconeogenic substrate, and DHAP serves as a metabolic branch point linking glycolysis, gluconeogenesis, and the GPS.

Fate 2: Glycerol-3-Phosphate Shuttle (GPS). Predominant in brain and muscle (29), cytosolic cGPDH catalyzes the reaction DHAP + cNADH + H⁺ ⇌ G3P + cNAD⁺ (30). NADH here may originate from glycolysis or from matrix substrates via the reverse MAS (rMAS) (31, 32). G3P is re-oxidized to DHAP by mitochondrial GPDH (mGPDH) on the inner membrane, generating FADH₂ for the ETC (reaction 7, Figure 3) (33). Each GPS cycle converts one cNADH to FADH₂, decreasing ATP yield by approximately one ATP (60% efficiency). Although seemingly inefficient under normal complex I (CI) function, under CI dysfunction plus KD, especially in high-energy tissues, this shuttle provides a valuable compensatory pathway for recovering energy from mNADH.

### 2.3 NADH and protein metabolism

NADH is intricately linked to protein metabolism via amino acid oxidation, neurotransmitter metabolism, and folate cycling (34).

Amino acid carbon skeletons enter the TCA cycle (35). Mitochondrial glutamate dehydrogenase (GDH) catalyzes oxidative deamination of glutamate (Glu) to *α*-ketoglutarate (*α*-KG) and mNADH, a key junction linking amino acid metabolism to the TCA cycle and energy state (36). Interconversions among *α*-KG, glutamate, glutamine (Gln), and *γ*-aminobutyric acid (GABA) critically influence neurotransmitter regulation (37). The folate (one-carbon) cycle, using tetrahydrofolate (THF) derivatives as one-carbon carriers (38), directly generates mNADH via the glycine cleavage system (GCS) (39). This mNADH can enter the ETC for ATP production. The folate cycle links amino acid metabolism, nucleotide synthesis, methylation, and NADPH generation, which are crucial for cell proliferation, epigenetics, and antioxidant defense (40). Under CI dysfunction, mNADH accumulation may inhibit GCS, impacting NADPH generation via the folate cycle (41).

### 2.4 TCA cycle flux and the NADH accumulation paradox

Under normal CI function, the TCA cycle serves as a hub for generating various metabolites, mNADH, and FADH₂. A core CI function is oxidizing mNADH to mNAD⁺, which is essential for sustaining TCA cycle enzyme activity. However, under CI dysfunction, reliance on glucose as the primary fuel maintains high insulin levels, inhibiting fatty acid mobilization and protein breakdown, thus depriving the TCA cycle of anaplerotic substrates. Elevated NADH/NAD⁺ ratios directly inhibit PDH, IDH3, and *α*-KGDH, blocking carbon flow at the entry and mid-stages of the TCA cycle. Furthermore, the high NADH/NAD⁺ ratio forces mMDH to run in reverse (OAA + mNADH → Malate + mNAD⁺), depleting oxaloacetate (OAA) and removing the acceptor for acetyl-CoA. These four factors collectively cause TCA cycle stagnation due to accumulated mNADH that cannot be re-oxidized to mNAD⁺ via CI, highlighting the need for a systemic solution to oxidize and re-utilize accumulated mNADH through alternative pathways.

## 3 Metabolic reprogramming of NADH under CI dysfunction and ketogenic diet

The core principle of the KD lies in “dredging, not repairing”—not attempting to fix the structurally damaged CI, but rather diverting accumulated mNADH into other utilizable forms via multiple metabolic pathways (“outflow”). This metabolic reprogramming mitigates the positive-feedback vicious cycle caused by CI dysfunction. It involves intra-matrix conversion of NADH to NADPH (Pathway 1, Figure 4) and BHB (Pathway 2); export of mNADH to the cytosol as malate via rMAS, regenerating cNADH; and cytosolic cNADH re-routing for energy recovery via FADH₂ (Pathway 3), or cytosolic malate utilization for gluconeogenesis (Pathway 4), cytosolic NADPH generation (Pathway 5).

**Figure 4:**
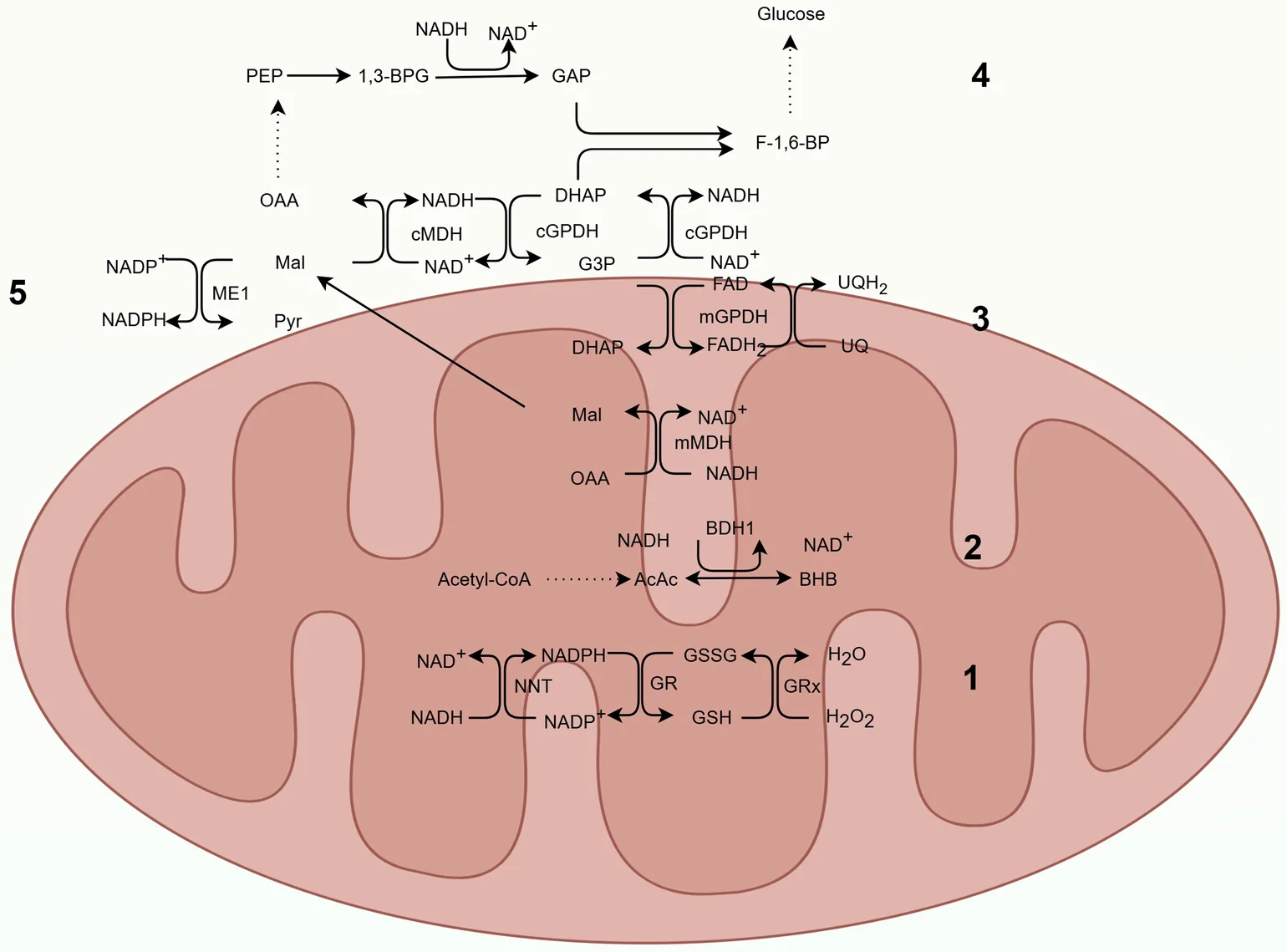
Metabolic reprogramming of NADH under CI dysfunction and KD. 1,3-BPG, 1,3-bisphosphoglycerate; GAP, glyceraldehyde 3-phosphate; F-1,6-BP, fructose-1,6-bisphosphate; DHAP, dihydroxyacetone phosphate; cGPDH, cytoplasmic glycerol-3-phosphate dehydrogenase; mGPDH, mitochondrial glycerol-3-phosphate dehydrogenase; cMDH, cytosolic malate dehydrogenase; mMDH, mitochondrial malate dehydrogenase; OAA, oxaloacetic acid; ME1, malic enzyme 1; G3P, glycerol 3-phosphate; Mal, malic acid; Pyr, pyruvate; PEP, phosphoenolpyruvate; KDs, ketogenic diets; NNT, NAD**(P)**⁺ transhydrogenase; UQH₂, reduced ubiquinone; UQ, oxidized ubiquinone; GR, glutathione reductase; GSH, glutathione; GSSG, glutathione disulfide; GRx, glutaredoxin; BHB, *β*-hydroxybutyrate; AcAc,acetoacetate.

### 3.1 Reducing equivalent transfer within the mitochondrial matrix

Form 1: NADH → NADPH. NADH and NADPH differ structurally by a phosphate group at the 2'-hydroxyl of the adenine ribose, dictating their distinct functions: NADH is primarily involved in energy metabolism, while NADPH functions in anabolism and antioxidant defense (34). Similar to NADH, we distinguish cytosolic NADPH (cNADPH), cytosolic NADP⁺ (cNADP⁺), matrix NADPH (mNADPH), and matrix NADP⁺ (mNADP⁺).

Under CI dysfunction, ETC blockade and mNADH accumulation exacerbate ROS generation and oxidative stress. This prompts the inner mitochondrial membrane protein NAD(P)⁺ transhydrogenase (NNT; containing FAD) to catalyze the reduction of mNADP⁺ to mNADPH using mNADH, coupled with H⁺ pumping into the intermembrane space (Pathway 1, Figure 4) (6, 42). Simultaneously, matrix isocitrate dehydrogenase 2 (IDH2) and malic enzyme 3 (ME3) may also generate mNADPH (43, 44). NADPH is vital for maintaining glutathione reductase (GR) and thioredoxin reductase (TrxR) activities, which are crucial for scavenging ROS in dysfunctional mitochondria and enhancing antioxidant capacity (34, 45).

Form 2: Ketogenesis and Inter-organ Transport. During fasting or strict carbohydrate restriction (KD), hepatic FAO generates abundant acetyl-CoA (46). To maintain blood glucose, cytosolic gluconeogenesis consumes OAA, making OAA relatively insufficient compared to acetyl-CoA. The excess acetyl-CoA in hepatocytes is diverted to ketone bodies: *β*-hydroxybutyrate (BHB), acetoacetate (AcAc), and acetone (Pathway 2, Figure 4). *β*-hydroxybutyrate dehydrogenase 1 (BDH1) catalyzes the reversible, substrate concentration-driven reaction: AcAc + mNADH + H⁺ ⇌ BHB + mNAD⁺ (19). Hepatic ketogenesis arises from reduced TCA flux (consumption < generation) and simultaneously provides a pathway to drain accumulated mNADH. Ketones are transported via monocarboxylate transporters (MCTs) from the liver to extrahepatic tissues (brain, heart, kidney) through the blood (47). These tissues possess robust ketolytic enzymes, reconverting ketones to acetyl-CoA for complete oxidation via the TCA cycle (48).

### 3.2 NADH/NAD⁺ ratio gradients and transmembrane NADH transfer

The NADH/NAD⁺ ratio reflects cellular redox state and regulates glycolysis, FAO, amino acid catabolism, and TCA cycle flux to balance energy supply and demand (49). In CI dysfunction, abnormal matrix and cytosolic NADH/NAD⁺ ratios are core indicators of an energy crisis (50).

According to the Nernst equation, the direction of the malate-aspartate shuttle (MAS) is driven by transmembrane concentration gradients of its substrates, including the NADH/NAD⁺ ratio gradient (51, 52). Under CI dysfunction plus KD, the cytosol becomes relatively oxidized due to suppressed glycolysis (from low glucose) and activated gluconeogenesis (3). The matrix becomes highly reduced due to blocked mNADH oxidation. This establishes a thermodynamic gradient favoring reverse MAS (rMAS) operation (53, 54). Physiologically, when the matrix NADH/NAD⁺ ratio exceeds the cytosolic ratio, rMAS is driven (52). In CI dysfunction plus KD, the matrix ratio can reach approximately 100-fold higher than the cytosolic ratio, strongly driving rMAS (3, 55, 56).

Reverse MAS (rMAS) Steps (Figure 5): In the matrix, OAA + mNADH → Malate + mNAD⁺ (mMDH). Malate exits via the OGC (oxoglutarate/malate carrier) in exchange for *α*-KG entering the matrix. In the cytosol, Malate + cNAD⁺ → OAA + cNADH (cMDH). OAA and glutamate are transaminated by cytosolic AST (aspartate aminotransferase) to aspartate (Asp) and *α*-KG. Aspartate enters the matrix via the AGC (aspartate/glutamate carrier) in exchange for glutamate exiting to the cytosol. In the matrix, Asp and *α*-KG are reconverted to OAA and glutamate by matrix AST, completing one rMAS cycle and achieving mNADH → cNADH conversion (56, 57).

**Figure 5:**
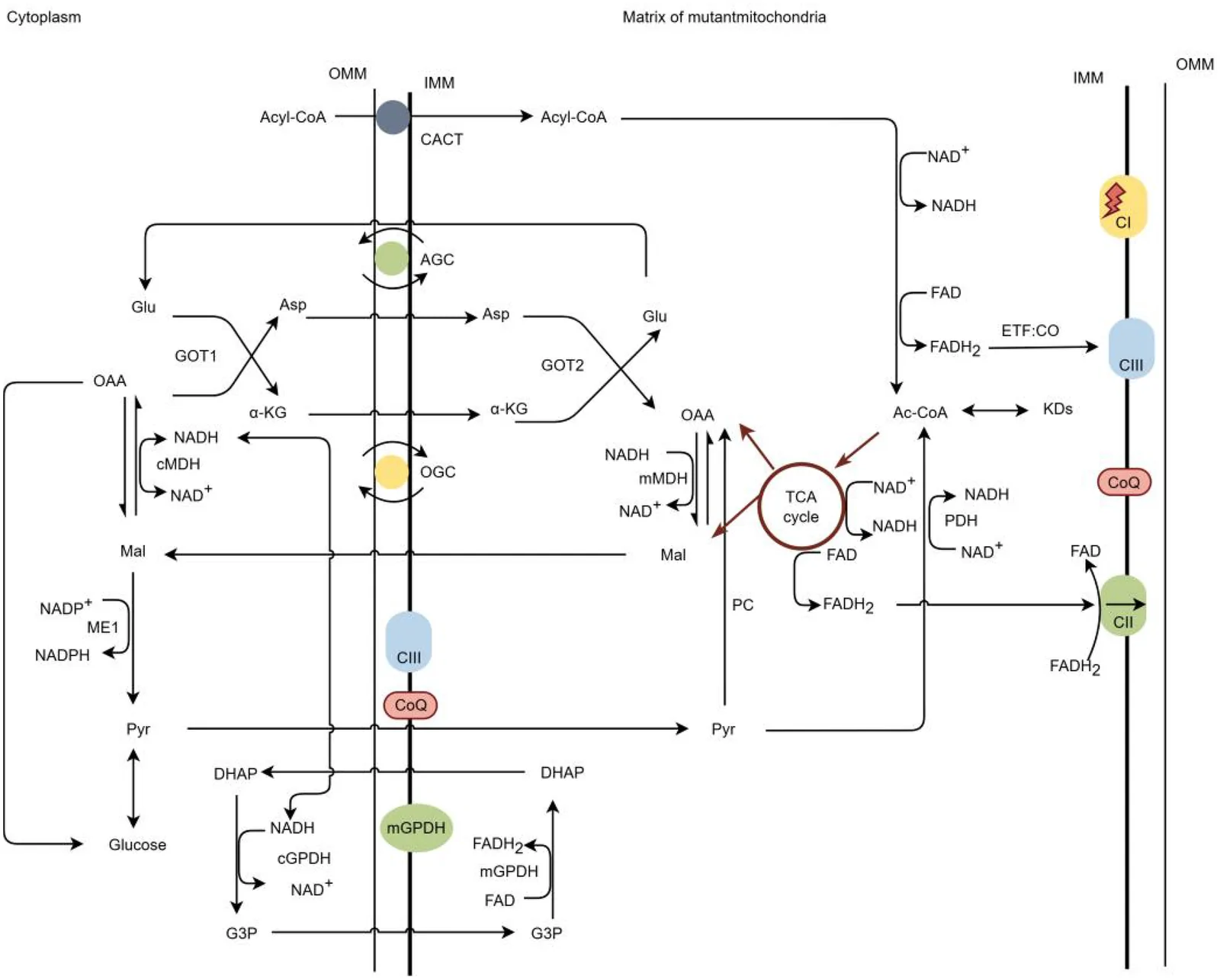
Metabolite generation and consumption in NADH reducing equivalent transfer across compartments. OMM, outer mitochondrial membrane; IMM, inner mitochondrial membrane; Acyl-CoA, fatty acyl-CoA; Ac-CoA, acetyl-CoA; CACT, carnitine-acylcarnitine translocase; ETF:QO, electron-transferring flavoprotein-ubiquinone oxidoreductase; KDs, ketone bodies; PDH, pyruvate dehydrogenase; AGC, aspartate-glutamate carrier; Glu, glutamic acid; *α*-KG, *α*-ketoglutaric acid; Asp, aspartate; PC, pyruvate carboxylase; GOT1, glutamic-oxaloacetic transaminase 1; GOT2, glutamic-oxaloacetic transaminase 2; OAA, oxaloacetic acid; Mal, malic acid; Pyr, pyruvate; cMDH/MDH1, cytosolic malate dehydrogenase; mMDH/MDH2, mitochondrial malate dehydrogenase; OGC, oxoglutarate/malate carrier; ME1, malic enzyme 1; DHAP, dihydroxyacetone phosphate; cGPDH, cytoplasmic glycerol-3-phosphate dehydrogenase; G3P, glycerol-3-phosphate; mGPDH, mitochondrial glycerol-3-phosphate dehydrogenase; CoQ, coenzyme Q; CI, complex I/NADH:ubiquinone oxidoreductase; CII, complex II/succinate dehydrogenase; CIII, complex III/ubiquinol-cytochrome c oxidoreductase.

By “pumping” mNADH out to the cytosol as cNADH via rMAS, the primary electron donor for the dysfunctional mitochondrial ETC shifts from mNADH to FADH₂ (supplied by CII, ETF:QO, mGPDH, etc.). Concurrently, the cytosolic reducing power source shifts from glycolytic cNADH to mNADH-derived cNADH via rMAS.

Tissues with high MAS expression (heart, liver, kidney) possess the greatest NADH export capacity under KD. The cNADH regenerated in the cytosolic pool from rMAS-transported malate can then be directed to various fates depending on tissue specificity and physiological needs.

### 3.3 Cytosolic fate 1: NADH → FADH₂ energy recovery pathway

cNADH can re-enter the mitochondrial intermembrane space via the GPS, being converted to FADH₂ to recover partial energy (Pathway 3, Figure 4) (33). Sustained GPS operation requires continuous supply of its substrates (DHAP and cNADH) and the ability to transfer FADH₂ electrons to UQ. Under CI dysfunction and KD, these conditions are met (58): rMAS provides cNADH via malate oxidation (31, 32); TAG-derived glycerol and active cytosolic gluconeogenesis supply DHAP; and the energy crisis creates a moderately oxidized ubiquinone pool. This pathway achieves ‘mNADH → cNADH → FADH₂ → UQ → CIII’ electron flow (Figure 5). Although less efficient (60% ATP yield) than normal, in tissues with high GPDH expression (fast-twitch muscle, brown fat, astrocytes), this serves as a vital compensatory mechanism during an energy crisis (15).

### 3.4 Cytosolic fate 2: gluconeogenesis

Matrix OAA (derived from lactate, pyruvate, or amino acid carbon skeletons under physiological conditions) can be converted to malate by mMDH and exported to the cytosol via rMAS, simultaneously transferring mNADH reducing equivalents (59). Cytosolic OAA, regenerated from malate via cMDH, is converted by phosphoenolpyruvate carboxykinase (PEPCK-C), consuming GTP, to phosphoenolpyruvate (PEP), which enters gluconeogenesis (Pathway 4, Figure 4) (60). Under CI dysfunction plus KD, with restricted carbohydrate intake, the OAA → malate → gluconeogenesis pathway is crucial: it maintains blood glucose, reduces mNADH accumulation, and facilitates recovery of TCA cycle flux.

### 3.5 Cytosolic fate 3: cNADPH generation

Cytosolic NADPH is essential for reductive biosynthesis (fatty acids, cholesterol, nucleotides) (7). Under CI dysfunction and KD, cytosolic malic enzyme 1 (ME1), which catalyzes the reaction Malate + cNADP⁺ → Pyruvate + CO₂ + cNADPH (Pathway 5, Figure 4), may contribute to cNADPH generation (61).

Under CI dysfunction and KD, removal of the high-insulin brake on lipolysis and proteolysis enables the following: matrix conversion of mNADH to mNADPH; consumption of mNADH via ketogenesis; and export of mNADH via rMAS-mediated malate transfer. These processes relieve allosteric inhibition of PDH, IDH3, and *α*-KGDH. Concurrently, FAO and amino acid catabolism replenish TCA cycle intermediates, restoring TCA flux and creating conditions to alleviate the energy crisis caused by CI dysfunction.

## 4 Discussion

The core of the “dredging rather than repairing” metabolic wisdom proposed in this article is that the efficacy of KD in treating CI-deficient mitochondrial diseases does not arise from repairing or simply bypassing the defective CI, but rather from the multi-pathway coordinated shunting, conversion, and reutilization of accumulated mNADH, thereby relieving allosteric inhibition of key TCA cycle enzymes, restoring TCA flux, and creating conditions for the establishment of FADH₂ compensatory pathways. Although the traditional “bypassing CI” view can explain the partial maintenance of electron flow, it cannot answer where the accumulated mNADH goes and therefore cannot explain how TCA cycle flux is restored. In fact, in CI deficiency, the hyperinsulinemic milieu induced by a high-carbohydrate diet suppresses lipolysis and proteolysis, depriving the TCA cycle of external anaplerotic substrate supply; simultaneously, CI is the primary entry point for mNADH oxidation, and bypassing it cannot consume the mNADH accumulated in the matrix. The high NADH/NAD⁺ ratio directly inhibits PDH, IDH3, and *α*-KGDH, blocking carbon flux at the entry and intermediate segments of the TCA cycle, while forcing reverse operation of mMDH, which depletes oxaloacetate and deprives the TCA cycle of the substrate required to accept acetyl-CoA. These four factors together lead to TCA cycle stagnation; at this point, even if the CII pathway remains patent, the source of its substrate, succinate, has already been depleted, and the FADH₂ compensatory pathway likewise cannot operate. Therefore, NADH dredging is a prerequisite for the establishment of the FADH₂ compensatory pathway.

The dredging of mNADH is not a single reaction but a hierarchically organized synergistic network: (1) Immediate intramitochondrial conversion: NNT, IDH2, and ME3 catalyze the generation of mNADPH from mNADH/mNADP⁺ (6, 42–44), directly relieving matrix reductive pressure and providing reducing power for GR and TrxR to counteract ROS (34, 45); (2) Transmembrane efflux: when the matrix NADH/NAD⁺ ratio far exceeds that on the cytosolic side (up to 100-fold under CI deficiency and KD), rMAS is driven in reverse by the thermodynamic gradient (3, 55, 56), exporting mNADH to the cytosol using malate as a carrier and converting it to cNADH, thereby achieving a switch in the principal electron donor—the electron donor of the mitochondrial ETC switches from mNADH to FADH₂ (derived from CII, ETF:QO, and mGPDH), while the cytosolic reducing power switches from glycolytically produced cNADH to cNADH provided by rMAS; (3) Cytosolic reutilization: cNADH is converted to FADH₂ via the *α*-glycerophosphate shuttle to recover energy (33, 58), or is used in gluconeogenesis to maintain blood glucose and alleviate mNADH accumulation (59, 60), or generates cNADPH via ME1 to support reductive biosynthesis (61). These three levels of pathways act synergistically to form a complete “dredging network,” the initiating condition of which is that KD relieves the high-insulin-mediated inhibition of lipolysis and proteolysis, thereby providing external anaplerotic substrates for the TCA cycle.

This framework is highly consistent with clinical observations: retrospective multicenter studies have shown that KD effectively controls seizures and stabilizes neurological function in patients with CI deficiency(1, 2), and case reports confirm that the plasma lactate/pyruvate ratio decreases significantly after KD intervention(3), suggesting activation of the rMAS efflux → cytosolic NADH reutilization pathway. KD is better tolerated in patients with CI deficiency than in those with other respiratory chain complex defects(1), possibly because patients with CI deficiency have more pronounced matrix NADH accumulation and thus benefit more from rMAS efflux; ketone bodies (BHB, AcAc) themselves also consume mNADH via the reversible reaction catalyzed by BDH1 (19), providing direct evidence for immediate intramitochondrial conversion. In addition, the therapeutic effect may be tissue-specific: tissues with high rMAS expression (heart, liver, kidney) have the greatest mNADH efflux capacity (56, 57), whereas tissues with high *α*-glycerophosphate shuttle expression (fast-twitch muscle fibers, brown adipose tissue, astrocytes) have a stronger capacity for cNADH→FADH₂ energy recovery (33, 58). This may explain interindividual variability in KD efficacy, and future studies should evaluate the expression of key enzymes in these pathways across different tissues in patients to predict responsiveness.

This framework remains a hypothesis, and its validation requires the following key experiments: (1) in CI-deficient cells or animal models, use ^13^C-labeled malate/aspartate to track transmembrane flux, or use genetically encoded NADH/NAD⁺ fluorescent probes to monitor dynamic changes in compartmental ratios; (2) use tissue-specific knockout mice to compare the degree of dependence on dredging pathways in tissues such as heart, liver, and brain; (3) detect changes in the activity of key enzymes in rMAS and the *α*-glycerophosphate shuttle in peripheral blood or muscle biopsies from patients with CI deficiency before and after KD intervention, and perform correlation analysis with clinical efficacy indicators. In addition, the quantitative characteristics of the new steady state of mNADPH generation (via NNT, IDH2, and ME3) and consumption (via GR and TrxR) under CI deficiency and KD remain unclear and need to be elucidated by combining metabolic flux analysis and redox proteomics.

In summary, the “dredging rather than repairing” metabolic wisdom proposed in this article elevates the mechanism of KD intervention in CI-deficient mitochondrial diseases from passive compensation by “bypassing the defect” to active reprogramming by “dredging the accumulation.” The core logic is that NADH dredging is a prerequisite for the establishment of the FADH₂ compensatory pathway, and elucidating the NADH dredging pathway is key to explaining TCA cycle restart. This framework provides a unified metabolic explanation for understanding the efficacy of KD and offers a new theoretical basis and testable predictions for individualized metabolic therapy of CI-deficient mitochondrial diseases.

## Data Availability

The original contributions presented in the study are included in the article/Supplementary Material, further inquiries can be directed to the corresponding author.
